# NUF2通过调控铁死亡促进NSCLC进展的机制研究

**DOI:** 10.3779/j.issn.1009-3419.2026.106.23

**Published:** 2026-08-20

**Authors:** Yani LIU, Jiaxuan REN, Ruibin ZHAO, Xiaoru MA, Yunbo DI, Yirong XU, Zhenwen CHEN

**Affiliations:** 1 030001 太原，山西医科大学基础医学院; 1 College of Basic Medical Sciences, Shanxi Medical University, Taiyuan 030001, China; 2 032200 汾阳，山西医药学院基础医学院; 2 College of Basic Medical Sciences,Shanxi University of Medicine, Fenyang 032200, China; 3 032200 汾阳，山西省汾阳医院; 3 Fenyang Hospital of Shanxi, Fenyang 032200, China

**Keywords:** 肺肿瘤, NUF2, SLC7A11, ACSL4, 铁死亡, Lung neoplasms, NUF2, SLC7A11, ACSL4, Ferroptosis

## Abstract

**背景与目的:**

NUF2（NUF2 component of NDC80 kinetochore complex）是确保染色体正确分离的核心调控蛋白，其异常表达与癌症的发生、发展密切关联。NUF2在非小细胞肺癌（non-small cell lung cancer, NSCLC）中表达水平高于正常组织，其在NSCLC中的作用机制尚未明确。本研究旨在探究NUF2促进NSCLC增殖、迁移和侵袭的作用机制。

**方法:**

GEPIA（Gene Expression Profiling Interactive Analysis）数据库分析NUF2在NSCLC组织与正常组织中的表达差异以及NUF2表达水平与患者总生存期的关系；通过免疫组化染色检测NSCLC组织中NUF2的阳性表达率并分析其与患者临床病理特征之间的关系。在A549细胞系中建立*NUF2*敲低和过表达稳转株，通过蛋白质免疫印迹（Western blot, WB）和实时荧光定量聚合酶链式反应（quantitative real-time polymerase chain reaction, RT-qPCR）验证转染效率；采用细胞增殖与毒性检测试剂盒（cell counting kit-8, CCK-8）、平板克隆实验、划痕实验和Transwell实验分别检测转染后细胞的增殖、迁移和侵袭的情况；通过WB和RT-qPCR检测调控铁死亡相关蛋白SLC7A11和ACSL4的表达水平；相应试剂盒检测丙二醛（malondialdehyde, MDA）、活性氧（reactive oxygen species, ROS）、亚铁离子（ferrous ions, Fe^2+^）和还原型谷胱甘肽（reduced glutathione, GSH）的含量，透射电镜（transmission electron microscopy, TEM）观察细胞内线粒体数量和结构变化。建立异种移植瘤动物模型，WB、RT-qPCR和免疫组化检测NUF2、SLC7A11和ACSL4的表达情况。

**结果:**

GEPIA数据库分析结果显示NUF2在NSCLC中显著高表达（*P*<0.05），并与患者总生存期呈负向关联（*P*<0.001）。免疫组化统计结果表明，NUF2高表达与患者的临床分期高（*P*<0.05）和淋巴结转移（*P*<0.01）有关。*NUF2*敲低后NSCLC的增殖、迁移、侵袭能力降低（*P*<0.001），移植瘤体积减小（*P*<0.05），*NUF2*敲低后调节铁死亡的关键分子SLC7A11表达降低（*P*<0.01）、ACSL4则升高（*P*<0.01），MDA（*P*<0.01）、ROS以及细胞Fe^2+^含量（*P*<0.001）均升高，而GSH含量降低（*P*<0.001），TEM观察发现敲低*NUF2*使线粒体缩小、嵴减少和膜密度增加。过表达*NUF2*后NSCLC的增殖、迁移、侵袭能力升高（*P*<0.001），移植瘤体积增大（*P*<0.001），SLC7A11表达升高（*P*<0.01）、ACSL4则降低（*P*<0.01），ROS（*P*<0.001）、MDA以及细胞Fe^2+^含量均降低（*P*<0.01），GSH含量升高（*P*<0.01）。

**结论:**

NUF2在NSCLC中高表达与患者预后差及易发生淋巴结转移密切关联，NUF2可能通过上调SLC7A11和下调ACSL4的表达抑制铁死亡，从而促进NSCLC的增殖、迁移、侵袭。

## Abstract (en)

**Background and objective:**

NUF2 component of NDC80 kinetochore complex (NUF2) is a core regulatory protein that ensures proper chromosome segregation, and its aberrant expression is closely associated with cancer initiation and progression. Although the expression level of NUF2 is higher in non-small cell lung cancer (NSCLC) than in normal tissues, its mechanism of action in NSCLC remains unclear. This study aims to investigate the mechanism by which NUF2 promotes the proliferation, migration, and invasion of NSCLC.

**Methods:**

The expression differences of NUF2 between NSCLC and normal lung tissues as well as the correlation between NUF2 expression and overall survival (OS) of NSCLC patients were analyzed via the Gene Expression Profiling Interactive Analysis (GEPIA) database. Immunohistochemical (IHC) staining was performed to detect the positive expression rate of NUF2 in NSCLC specimens. NUF2 knockdown and overexpression stable cell lines were established in the A549 cell line, and transfection efficiency was verified by Western blot (WB) and quantitative real-time polymerase chain reaction (RT-qPCR). The proliferation, migration, and invasion of transfected cells were assessed using the cell counting kit-8 (CCK-8), colony formation assay, wound healing assay, and Transwell assay. The expression levels of ferroptosis-related proteins were determined using WB and RT-qPCR. Commercial assay kits were utilized to measure the contents of malondialdehyde (MDA), reactive oxygen species (ROS), ferrous ion (Fe²^+^) and glutathione (GSH), while intracellular mitochondrial structural alterations were visualized by transmission electron microscopy (TEM). Xenograft tumor models were established. The expression levels of NUF2, SLC7A11 and ACSL4 were detected by WB, RT-qPCR and IHC.

**Results:**

GEPIA analysis revealed that NUF2 was significantly highly expressed in NSCLC (*P*<0.05) and was associated with OS (*P*<0.001). Clinical statistical analyses demonstrated that upregulated NUF2 was significantly associated with advanced clinical stage (*P*<0.05) and lymph node metastasis (*P*<0.01). NUF2 knockdown markedly suppressed the proliferation, migration and invasion of NSCLC cells (*P*<0.001) and reduced xenograft tumor volume *in vivo* (*P*<0.05). Mechanistically, NUF2 silencing downregulated the expression of the ferroptosis master regulator SLC7A11 (*P*<0.01) while upregulating ACSL4 (*P*<0.01). The levels of MDA (*P*<0.01), ROS and Fe²^+^ (*P*<0.001) were all elevated following NUF2 knockdown, while the content of GSH decreased (*P*<0.001). TEM observation revealed that NUF2 knockdown led to mitochondrial shrinkage, reduced cristae number and increased membrane density. Conversely, NUF2 overexpression enhanced NSCLC cell proliferation, migration and invasion (*P*<0.001) and increased xenograft tumor volume (*P*<0.001). Meanwhile, SLC7A11 expression was elevated (*P*<0.01) and ACSL4 expression was decreased (*P*<0.01). The levels of ROS (*P*<0.001), MDA and Fe²^+^ were decreased (*P*<0.01), and the GSH level was increased (*P*<0.01).

**Conclusion:**

NUF2 is highly expressed in NSCLC and is closely associated with poor prognosis and lymph node metastasis. NUF2 inhibits ferroptosis by upregulating SLC7A11 expression and downregulating ACSL4 expression, thereby promoting the proliferation, migration, and invasion of NSCLC.

2022年全球癌症统计数据^[1]^显示，肺癌在全部恶性肿瘤中的新增病例数与死亡病例数均高居榜首，占全球新发癌症的近12.4%以及癌症死亡人数的18.7%。肺癌在我国发病率和死亡率也居首位^[2]^。尽管非小细胞肺癌（non-small cell lung cancer, NSCLC）的治疗手段取得了很大的进展，但由于NSCLC的高度异质性，晚期NSCLC患者的生存率仍然不理想，5年生存率不超过20%^[3]^。因此，进一步了解NSCLC发生和发展的分子机制，寻找更有效的分子治疗靶点，对提高晚期NSCLC患者的治疗和生存率具有重要意义。

有丝分裂是人体内细胞进行自我复制和分裂的核心过程，染色体分离作为有丝分裂过程中的关键步骤，其根本意义在于保证遗传物质分配的绝对精确性，从而维护遗传的稳定性。NUF2（NUF2 component of NDC80 kinetochore complex）又被称为细胞分裂相关蛋白1，其编码基因染色体定位于1q23.3，在细胞核和胞质中表达。NUF2能够在细胞分裂过程中使染色体正确分离^[4]^，保障遗传物质准确传递，维持基因组稳定性。NUF2在癌症中的核心机制是其异常表达使染色体不稳定，进而通过激活多个分子途径，最终促进肿瘤发生发展^[5]^。在肝细胞癌中抑制NUF2后癌细胞发生细胞周期停滞和细胞凋亡^[6]^。在胃癌中NUF2的高度表达与患者的生存期短及预后不良有关，并且NUF2转录水平与高免疫细胞浸润之间存在显著的负相关^[7]^。研究^[8]^发现，NUF2在肺腺癌中高表达，可充当促癌基因并作为NSCLC潜在的预后生物标志物，然而NUF2在NSCLC进展中所涉及的分子机制并未得到深入探索。

铁死亡（ferroptosis）是一种程序性细胞死亡方式，参与了多种生物代谢过程，包括氨基酸代谢、脂质代谢、铁代谢等。铁死亡的具体机制是亚铁离子（ferrous ions, Fe^2+^）将H_2_O_2_转化为剧毒的羟基自由基，攻击膜上的多不饱和脂肪酸（polyunsaturated fatty acids, PUFA），导致脂质过氧化反应发生。当细胞自身的抗氧化系统谷胱甘肽过氧化物酶4（glutathione peroxidase 4, GPX4）无法修复时，膜结构崩溃，细胞死亡^[9]^。在形态学方面，铁死亡的典型标志包括线粒体收缩和嵴减小或者缺失，同时伴随着膜密度的上升及外膜壁破损。此外，细胞核的大小保持稳定且没有染色质的凝固现象，而到了晚期阶段，细胞膜会出现破裂的现象。在生物化学特性方面，铁死亡的主要特点是铁离子累积、活性氧（reactive oxygen species, ROS）聚集以及脂质过氧化等。调控铁死亡涉及许多关键分子蛋白，其中长链脂酰辅酶A合成酶4（acyl-CoA synthetase long chain family member 4, ACSL4）主要负责将PUFA活化并将其整合进膜磷脂^[10]^，这一过程是细胞对铁死亡敏感性的关键决定因素。作为铁死亡的核心调控因子，ACSL4通过增强脂质过氧化和氧化应激促进各种癌症中的铁死亡，使其成为非凋亡细胞死亡途径的关键参与者^[11]^。溶质载体家族7成员11（solute carrier family 7 member 11, SLC7A11）可促进谷胱甘肽（glutathione, GSH）的合成，从而激活GPX4，抵抗铁死亡^[12]^。此外SLC7A11在多种癌组织中高表达，被认为是一种重要的致癌蛋白^[13]^。

NUF2与铁死亡的直接调控关系尚未明确，现有证据多为通路交叉与分子关联。比如，NUF2沉默可诱导癌细胞凋亡，而p53通路可同时调控凋亡与铁死亡^[14]^。在肝癌中NUF2富集于细胞周期通路，而铁死亡的诱导依赖细胞周期状态，两者可能存在细胞周期依赖的协同效应^[15]^。本研究旨在探索NUF2在NSCLC患者中的作用机制及临床意义，并通过体内外实验对关键结果进行初步验证，揭示NUF2是否通过调控铁死亡途径促进NSCLC的增殖、迁移和侵袭。

## 1 材料和方法

### 1.1 临床样本

从山西省汾阳医院收集的石蜡包埋的NSCLC组织208例，癌旁组织70例。所有患者术前均未接受任何抗肿瘤治疗，无免疫缺陷疾病、无重要器官衰竭、无其他恶性肿瘤。整理纳入病例的临床病理资料，涵盖患者年龄、性别、肿瘤分化程度、组织学分型、临床分级以及肿瘤原发灶-淋巴结-转移（tumor-node-metastasis, TNM）分期。构建8个组织芯片，分别包括腺癌3个、鳞癌3个和癌旁2个，后续检测患者组织中NUF2的阳性表达率。此外，随访在患者知情同意下进行，研究截止时间为2023年12月30日，主要观察指标为总生存期（overall survival, OS）。此研究已经过山西医科大学汾阳学院（现改名为山西医药学院，伦理申请时学校未改名）伦理委员会的审查和批准（No.2025082）。

### 1.2 免疫组化染色

组织芯片和动物组织切片进行染色。切片置于70 ^o^C烤箱烘烤，之后利用二甲苯完成脱蜡，再经过梯度乙醇实现水化处理。使用柠檬酸钠（中杉金桥，pH 6.0，ZLI-9065）进行热活化抗原修复。1% H_2_O_2_室温孵育以消除内源性过氧化物酶干扰，马血清封闭切片。使用一抗NUF2抗体（武汉三鹰，15731-1-AP）1:100于4 ^o^C过夜孵育。IgG二抗（中杉金桥，PV-6000）37 ^o^C孵育30 min，DAB试剂显色，苏木素复染细胞核，梯度乙醇脱水，二甲苯透明，中性树胶封片后置于光学显微镜下观察。以染色强度评分与阳性细胞比例评分的乘积判定表达水平：染色强度按0（无）、1（弱，黄色）、2（中，棕黄色）、3（强，棕褐色）计分。阳性细胞比例按0（0%）、1（1%-25%）、2（26%-50%）、3（51%-75%）、4（76%-100%）计分。以乘积值4为截断值，>4为高表达，≤4为低表达。由两位病理医师在双盲的情况下完成。

### 1.3 细胞培养

细胞用DMEM/F12培养（含10%胎牛血清），置于37^ o^C、5% CO_2_孵箱内。每天观察细胞状态，根据生长情况进行培养基更换。

### 1.4 转染

在A549细胞系中用慢病毒感染构建*NUF2*稳定敲低和过表达细胞株（*NUF2*过表达载体编号：GV492；*NUF2*敲低载体编号：GV493）。将A549细胞以每孔1×10^5^个细胞接种到6孔板中，加入终浓度为1×的HitransG病毒感染增强液以及适量病毒液（病毒体积=感染复数×细胞数目/病毒滴度）（吉凯基因，REVG005）。感染72 h后，继续在含2 μg/μL嘌呤霉素的培养液中筛选48 h，然后收集细胞通过实时荧光定量聚合酶链式反应（quantitative real-time polymerase chain reaction, RT-qPCR）和蛋白质免疫印迹（Western blot, WB）验证转染效率。

### 1.5 WB

待测样本收集在裂解液中，冰上裂解30 min。4^ o^C、12,000 rpm条件下离心20 min，吸取蛋白上清。采用二喹啉甲酸（bicinchoninic Acid, BCA）蛋白浓度测定试剂盒测定蛋白样本浓度，每泳道上样15 μg总蛋白，经SDS-PAGE凝胶电泳完成蛋白分离，随后转印至聚偏氟乙烯（polyvinylidene fluoride, PVDF）膜。室温下5%脱脂牛奶摇床封闭2 h。将膜与NUF2、SLC7A11（武汉三鹰，26864-1-AP）和ACSL4（武汉三鹰，22401-1-AP）的抗体于4^ o^C过夜孵育。第二天室温下二抗摇床孵育2 h，洗涤后使用ECL发光试剂对蛋白条带进行可视化。使用Image J软件分析。

### 1.6 RT-qPCR

使用Trizol（Invitrogen, 15596018CN）裂解细胞或组织提取总RNA并检测纯度和浓度。按PrimeScript RT试剂盒（Takara, RR047A）说明书逆转录得到cDNA。用TB Green Premix Ex Taq II、cDNA和引物进行扩增。引物如下：*NUF2*：F-5’-TGGAGACTCAGTTGACTGCCTG-3’，R-5’-ATTTGGTCCTCCAAGTTCAGGCT-3’；*SLC7A11*：F-5’-TGCCCAGATATGCATCGTCC-3’，R-5’-TCTTCTTCTGGTACAACTTCCAGT-3’；*ACSL4*：F-5’-GCCCACTTCAGACAAACCTGG-3’，R-5’-ACAGCTTCTCTGCCAAGTGTGG-3’；*GAPDH*：F-5’-CAGGAGGCATTGCTGATGAT-3’，R-5’- GAAGGCTGGGGCTCATTT-3’。采用2^−ΔΔCt^法计算相对基因表达。

### 1.7 细胞活力测定

每孔2×10^3^个转染细胞接种到96孔板，分别在接种细胞0、24、48、72 h后加入10 μL细胞增殖与毒性检测试剂盒（cell counting kit-8, CCK-8）溶液（索莱宝，CA1210）孵育1 h。450 nm处测定吸光度值。

### 1.8 平板克隆实验

将各组细胞按照800个/孔接种至6孔板，置于培养箱连续培养14 d，每间隔3 d更换一次完全培养基。培养结束后使用4%多聚甲醛固定细胞，0.1%结晶紫染液对细胞克隆进行染色。采集图像后用Image J软件分析统计各组克隆形成数目。

### 1.9 划痕实验

先在6孔板背面划线，将转染好的细胞以5×10^5^个/孔密度接种于6孔板，培养24 h后（细胞密度达到90%），垂直于孔板划直线，然后用磷酸盐缓冲液（phosphate-buffered saline, PBS）洗涤，换新鲜无血清或低浓度血清培养基培养。分别于划痕后0 h和24 h拍照。Image J软件分析迁移率。细胞迁移率为（0 h划痕面积-24 h划痕面积）/0 h划痕面积。

### 1.10 Transwell实验

将转染后的细胞用无血清培养基重悬成密度为2.5×10^5^个/mL的细胞悬液，接种到含有基质胶（迁移不用加基质胶，侵袭需要）的上室，下室加入含20% FBS的培养基，24孔板中培养48 h，4%多聚甲醛固定，0.1%结晶紫染色。拍照并通过Image J软件计算细胞数。

### 1.11 脂质过氧化检测

按照脂质氧化MDA检测试剂盒（碧云天，S0131S）进行操作。细胞中加入裂解液进行裂解，12,000 *g*，离心10 min后取上清。将上清与工作液按比例混匀后100^ o^C加热，然后1000 *g*，离心10 min后取上清。在532 nm波长处测定吸光度。

### 1.12 ROS检测

各组细胞按5×10^5^个/孔接种到6孔板，培养24 h后按1:1000将DCFH-DA（索莱宝，CA1410）用无血清培养基稀释后加入细胞中，避光孵育30 min。荧光显微镜下拍照检测。

### 1.13 Fe^2+^测定

按照细胞Fe^2+^红色荧光检测试剂盒（碧云天，S1070S）说明书进行操作。将细胞以5×10^5^个/孔接种到6孔板，培养24 h，将1000×的RhoNox-6（Fe^2+^红色荧光探针）和Hoechst 33342（细胞核蓝色荧光）用无血清培养基稀释到1×，细胞中加入染液，37 ^o^C孵育30 min。荧光显微镜下观察。

### 1.14 还原型GSH

按照还原型GSH（索莱宝，BC1175）说明书进行操作。细胞样本收集不少于10^6^个细胞，PBS清洗后加入试剂一（裂解）重悬，超声破碎细胞（功率200 w，超声3 s，间隔10 s，重复30次），12,000 *g*，4 ^o^C离心10 min后取上清，上清中加入试剂二（含DTNB的显色剂）和试剂三（辅助显色），混匀静置2 min，酶标仪读取412 nm处吸光度。

### 1.15 透射电镜（transmission electron microscopy, TEM）检测线粒体超微结构

收集各组待检测细胞，样本经过戊二醛-锇酸双重固定、梯度脱水、树脂包埋、超薄切片、重金属染色一系列处理。使用TEM观察细胞内部线粒体超微形态，对比不同组别线粒体外形、线粒体嵴数量以及膜电子密度差异。样本制备与电镜成像工作委托赛维尔生物公司协助完成。

### 1.16 动物实验

本研究中的12只BALB/c-裸鼠（5周龄，雄性）购自北京斯贝福生物技术有限公司[许可证号：SCXK（京）2024-0001]。随机分为4组，每组3只，饲养环境为温度25-26 ^o^C，明暗循环10 h/14 h，湿度为50%-60%。将3×10^6^个细胞注射到裸鼠右腋窝皮下。每3天监测1次小鼠体重，每7天测量1次肿瘤体积。在第45天处死小鼠，分离肿瘤并拍照。肿瘤体积（mm^3^）=长（mm）×宽^2^（mm^2^）×Π/6。所有动物实验均获得山西医科大学汾阳学院伦理委员会批准（No.2025082）。

### 1.17 统计分析

数据统计分析借助SPSS和GraphPad Prism。用*Kaplan-Meier*及*Log-rank*检验进行生存分析，计量资料用均数±标准差表示，两组及多组间比较分别采用t检验和单因素方差分析。所有实验独立重复3次，P<0.05为差异有统计学意义。

## 2 结果

### 2.1 NUF2在NSCLC组织中高表达，与疾病进展和预后有关

在GEPIA（Gene Expression Profiling Interactive Analysis）数据库查找NUF2在肺癌中的表达情况，表达差异分析图（附图1A， http://www.lungca.org/files/2026s7/2026s7-s1.pdf）显示，肿瘤组织的表达明显高于癌旁组织；*Kaplan-Meier*生存分析（附图1B， http://www.lungca.org/files/2026s7/2026s7-s1.pdf）结果显示，NUF2表达水平越高的患者OS越短。生存分析发现NUF2高表达的患者OS较低表达患者明显缩短（图1A）。免疫组化检测208例NSCLC患者组织中NUF2的表达情况，发现主要表达在细胞核（图1B），与70例癌旁组织样本进行比较，癌组织中阳性表达率（125/208, 60.1%）高于癌旁正常组织（15/70, 21.4%），差异有统计学意义（*P*<0.001）。NUF2表达与患者T分期和淋巴结转移有关，未观察到NUF2水平与性别、年龄和M分期有明显关联（表1）。

**图1:**
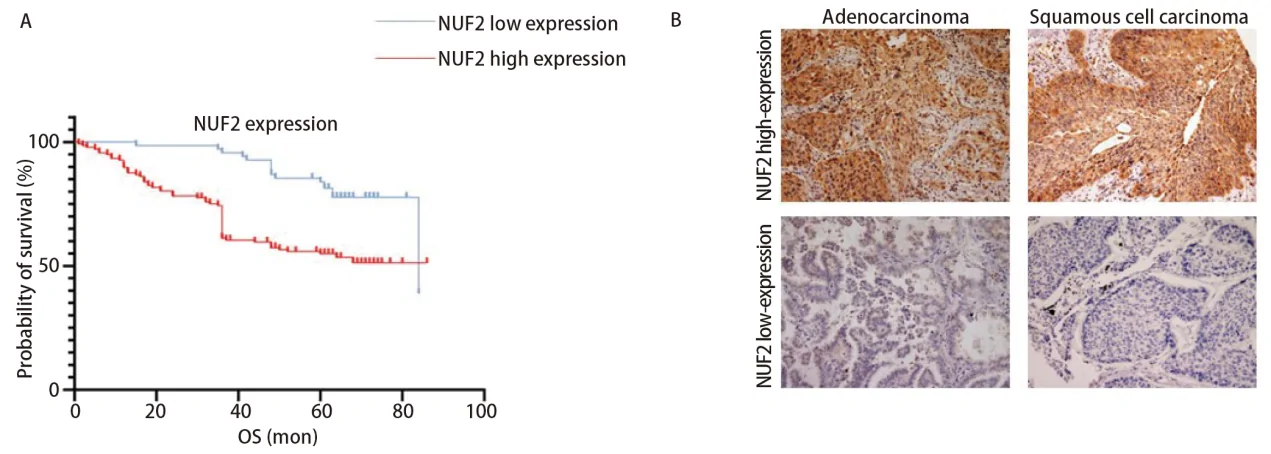
NUF2在肺癌中的表达及预后分析。A: NUF2表达水平与患者OS的Kaplan-Meier生存曲线（P<0.001）；B: NUF2在肺腺癌和肺鳞癌组织中的表达情况（IHC, ×200）。

**表1:** NSCLC患者NUF2表达与临床病理特征的关系（*n*=208）

Index		*n*	Low-expression, *n* (%)	High-expression, *n* (%)	*P*
Sex	Male Female	154 54	61 (39.6) 22 (40.7)	93 (60.4) 32 (59.3)	0.884
Age (yr)	≤60 >60	96 112	34 (35.4) 49 (43.8)	62 (64.6) 63 (56.2)	0.221
T stage	T1-T2 T3-T4	166 42	73 (44.0) 10 (23.8)	93 (56.0) 32 (76.2)	0.017
N stage	N0 N1-N2	131 77	62 (47.3) 21 (27.3)	69 (52.7) 56 (72.7)	0.004
M stage	M0 M1	204 4	81 (39.7) 2 (50.0)	123 (60.3) 2 (50.0)	0.677
Grade	1-2 3	85 123	37 (43.5) 46 (37.4)	48 (56.5) 77 (62.6)	0.375
Pathological type	Adenocarcinoma Squamous cell carcinoma	103 105	45 (43.7) 38 (36.2)	58 (56.3) 67 (63.8)	0.270

NSCLC: non-small cell lung cancer; TNM: tumor-node-metastasis.

### 2.2 NUF2促进肺癌细胞的增殖、迁移和侵袭

用慢病毒转染A549细胞系建立稳转细胞株，分别为KD-NC组（*NUF2*敲低对照组）和NUF2-KD组（*NUF2*敲低组）、OE-NC组（*NUF2*过表达对照组）和NUF2-OE组（*NUF2*过表达组）。使用WB和RT-qPCR检测慢病毒敲低和过表达*NUF2*后细胞中的蛋白和mRNA表达水平。与KD-NC组相比，NUF2-KD组的蛋白和mRNA表达水平分别下降了44%（*P*<0.01）和73%（*P*<0.001）（图2A、2B），NUF2-OE组的蛋白和mRNA水平分别是OE-NC组的1.7倍（P<0.01）和8.7倍（*P*<0.001）（图3A、3B）。采用CCK-8实验和平板克隆实验检测NUF2对肺癌细胞增殖的影响，结果表明，NUF2-KD组的细胞增殖能力与KD-NC组相比明显降低（*P*<0.001）（图2C、2D）；与OE-NC组相比，NUF2-OE组的细胞增殖能力明显增强（*P*<0.001，图3C、3D）。随后使用划痕实验和Transwell评估NUF2对A549细胞迁移和侵袭能力的影响。划痕实验结果显示，敲低*NUF2*后细胞迁移率由KD-NC组的（22.25±1.04）%显著下降至（12.18±0.28）%（*P*<0.001，图2E）；Transwell实验表明，与KD-NC组相比，NUF2-KD组穿过上室孔膜的细胞数由（236.67±11.24）个/视野减少至（93.33±6.66）个/视野（*P*<0.001），且穿过上室基质胶的细胞数由（180.67±5.13）个/视野减少至（52.33±3.51）个/视野（*P*<0.001，图2F）。*NUF2*过表达后结果相反（图3E、3F）。以上结果说明，NUF2可以促进NSCLC的增殖、迁移和侵袭能力。

**图2:**
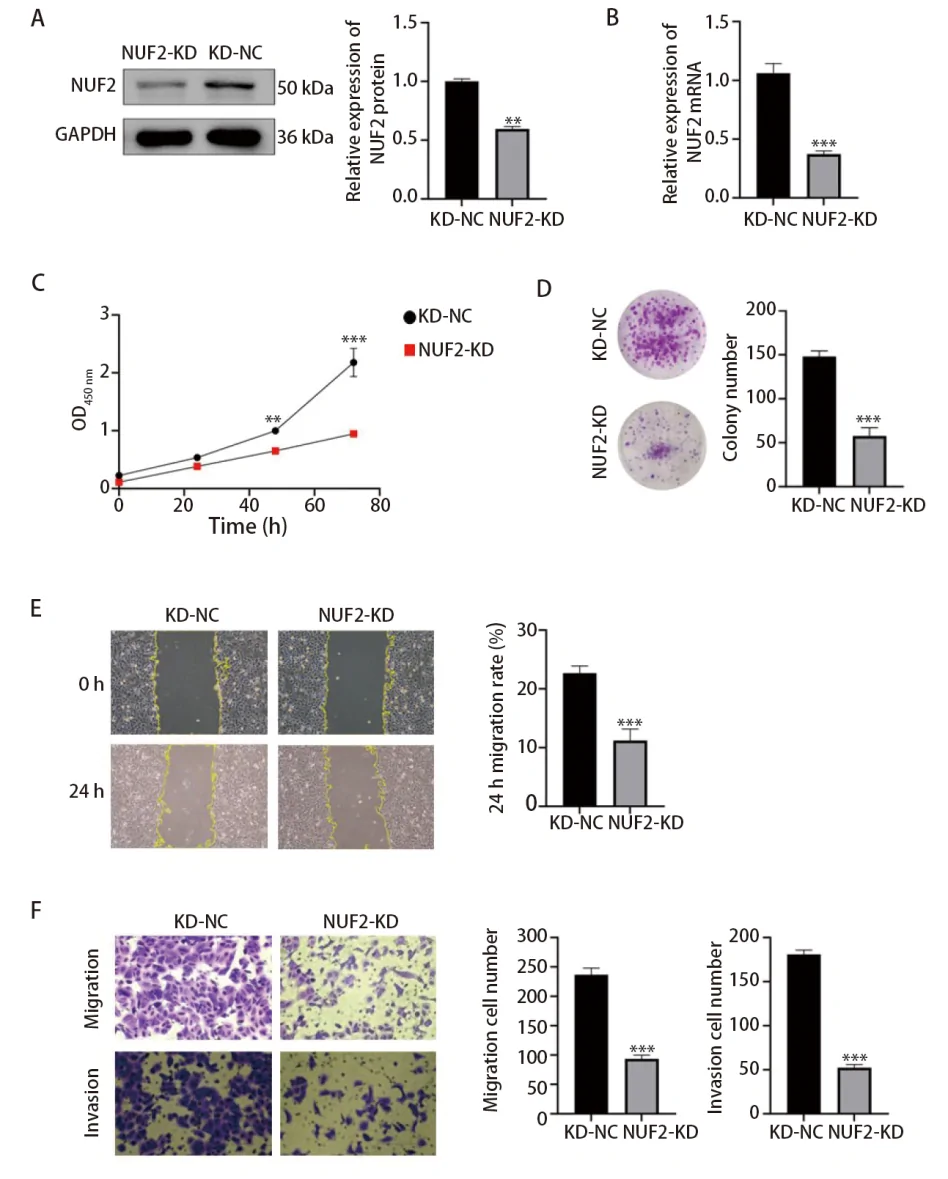
NUF2敲低对NSCLC细胞恶性表型的影响。A、B: WB和RT-qPCR验证NUF2敲低效率；C: CCK-8实验比较NUF2-KD组和KD-NC组的细胞活力；D: 平板克隆实验评估敲低NUF2对克隆形成能力的影响；E: 划痕实验检测敲低NUF2后细胞迁移率的变化；F: Transwell检测敲低NUF2对细胞迁移和侵袭能力的影响（结晶紫染色，×100）。

**图3:**
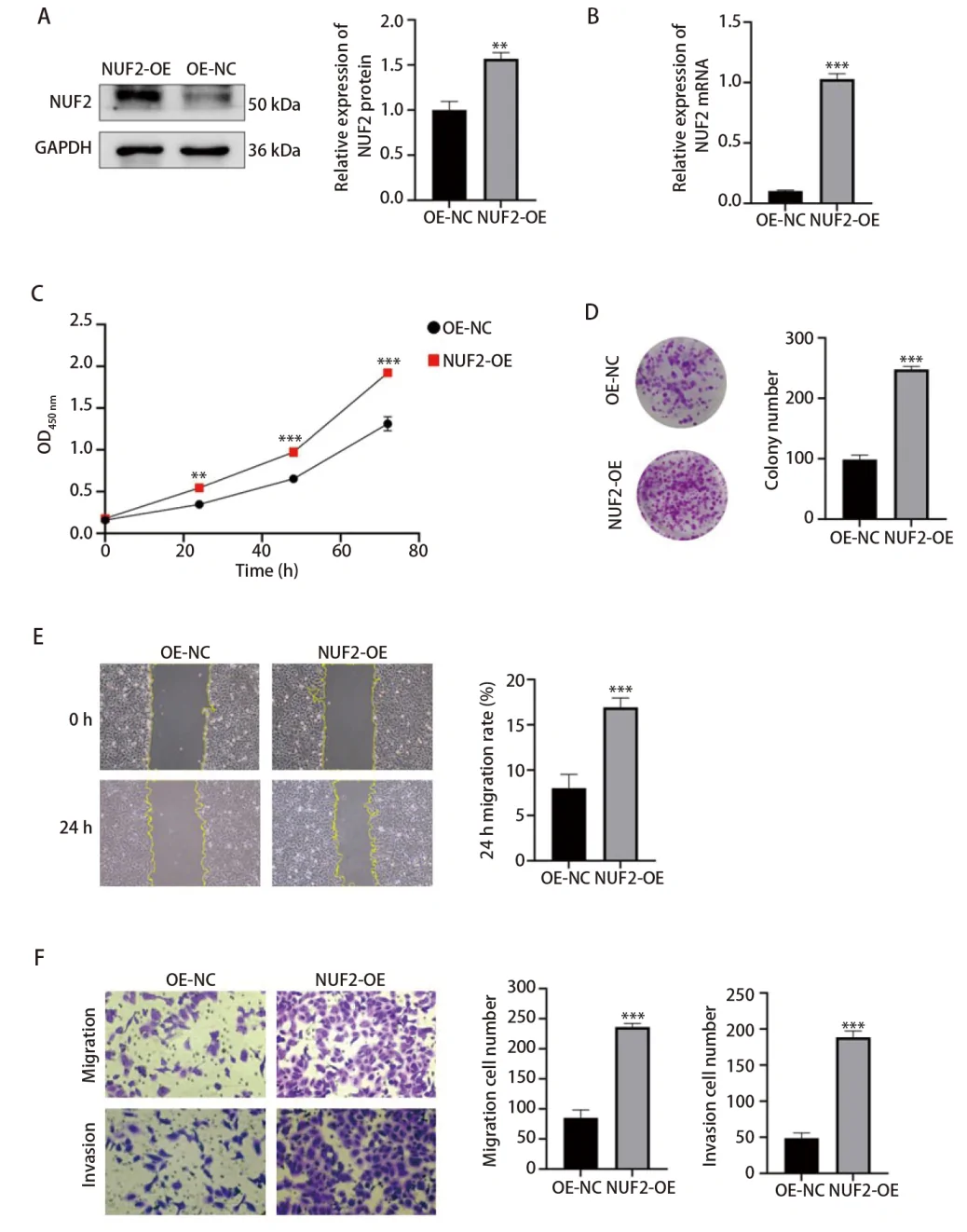
NUF2过表达对NSCLC细胞恶性表型的影响。A、B: WB和RT-qPCR验证NUF2过表达效率；C: CCK-8实验检测NUF2-OE组和OE-NC组的细胞活力差异；D: 平板克隆实验评估过表达NUF2对增殖能力的影响；E: 划痕实验比较过表达NUF2后细胞迁移率的变化；F: Transwell验证过表达NUF2对细胞迁移和侵袭能力的影响（结晶紫染色，×100）。

### 2.3 NUF2通过调控ACSL4和SLC7A11抑制NSCLC的铁死亡

ACSL4和SLC7A11是调控铁死亡的主要相关蛋白，线粒体结构改变、ROS、MDA、铁积累和GSH是发生铁死亡的重要指标。在构建*NUF2*敲低和过表达稳转细胞株中，通过WB和RT-qPCR检测铁死亡相关蛋白的表达情况。结果显示，与KD-NC组相比，NUF2-KD组SLC7A11的蛋白和mRNA表达水平均降低，过表达之后表达水平则升高（图4A、4C）；而*NUF2*敲低后ACSL4的蛋白和mRNA表达水平均升高，过表达*NUF2*后表达水平降低（图4B、4D）。以上结果表明，NUF2对ACSL4和SLC7A11的表达可能具有调控作用。

**图4:**
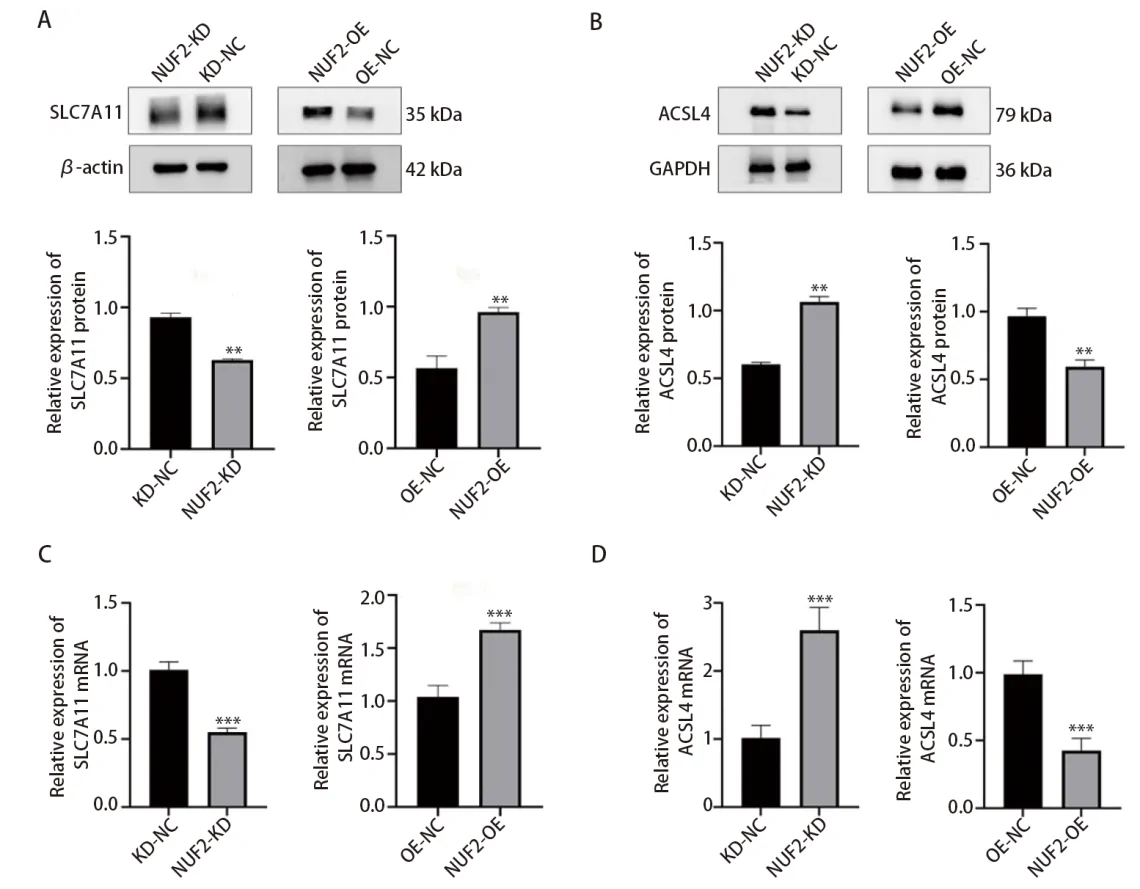
NUF2调控铁死亡相关蛋白SLC7A11及ACSL4的表达。A、B：WB检测敲低和过表达NUF2后SLC7A11和ACSL4的蛋白表达水平；C、D：RT-qPCR检测敲低和过表达NUF2后SLC7A11和ACSL4的mRNA表达水平。

TEM观察线粒体结构发现，与KD-NC组相比，NUF2-KD组线粒体缩小、线粒体内嵴部分减少甚至消失、膜密度增加（图5A）。脂质过氧化检测结果显示，NUF2-KD组的MDA含量高于KD-NC组，相反，NUF2-OE组的MDA含量明显低于OE-NC组（图5B）。通过DCFH-DA荧光探针检测ROS，敲低*NUF2*后，与KD-NC组相比，NUF2-KD组荧光强度平均值显著增加，而过表达*NUF2*后荧光强度平均值显著降低（图5C）。NUF2-KD组细胞内的Fe^2+^水平与KD-NC组相比显著升高，过表达后则相反（图5D）。此外，敲低*NUF2*后GSH的含量较KD-NC组相比显著减少，过表达后GSH含量增加（图5E）。以上这些发现表明NUF2高表达与抑制NSCLC细胞发生铁死亡有关。

**图5:**
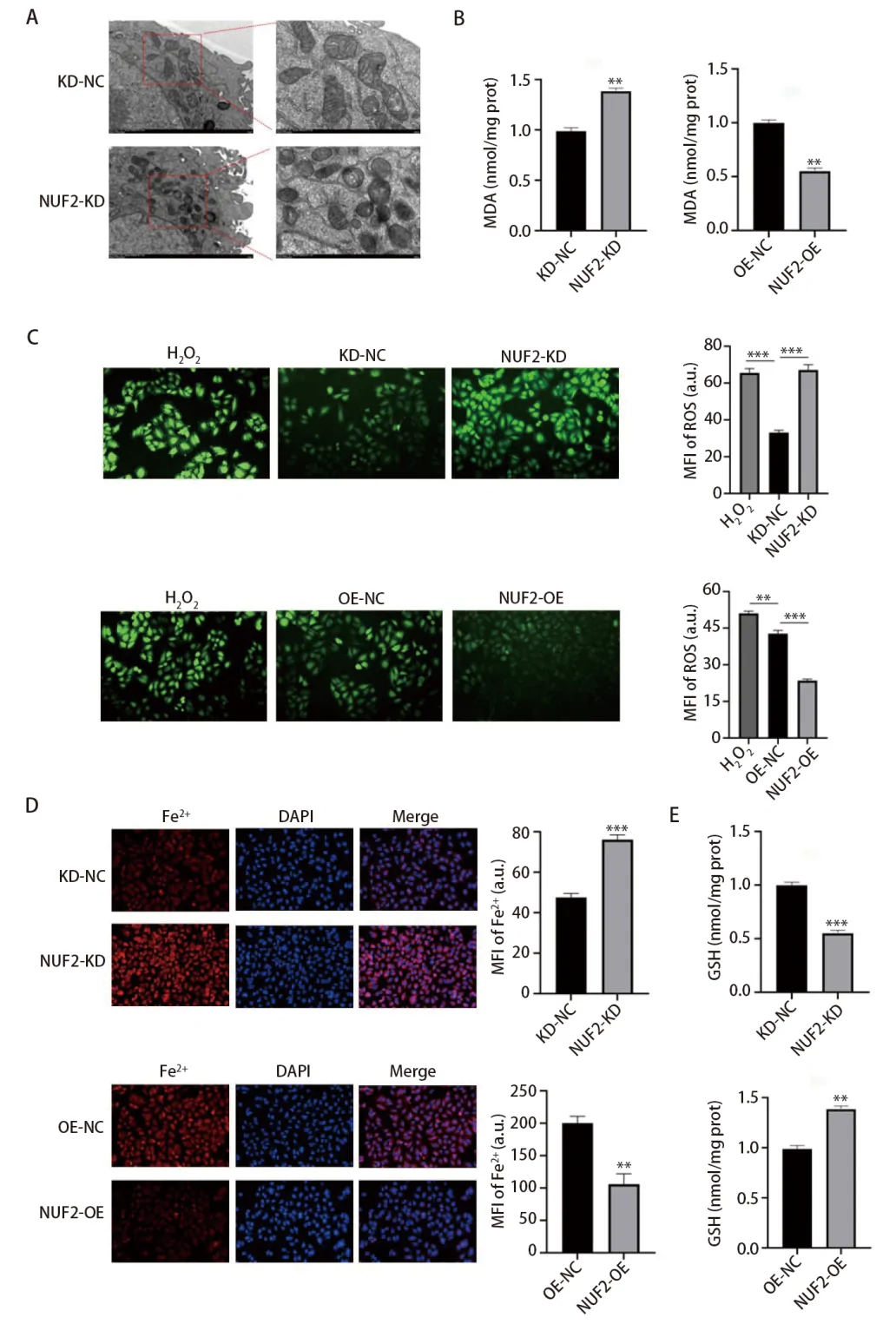
NUF2对NSCLC细胞铁死亡相关指标的影响。A: 对照组和NUF2敲低组的线粒体透射电镜图（×8000）；B：NUF2对细胞内MDA含量的影响；C：DCFH-DA染色检测NUF2对细胞内ROS水平的影响（×200）；D：FerroOrange染色检测NUF2对细胞内Fe^2+^水平的影响（×200）；E: NUF2对细胞内还原型GSH含量的影响。

### 2.4 铁死亡抑制剂逆转*NUF2*敲低所致细胞存活率与MDA含量变化的结果

CCK-8检测结果显示，与KD-NC组相比，NUF2-KD组细胞的存活率显著降低，表明*NUF2*敲低能够抑制细胞存活；在敲低*NUF2*的细胞中加入铁死亡抑制剂 Ferrostatin-1后细胞存活率升高（图6A），这说明铁死亡抑制剂能够有效逆转*NUF2*敲低所导致的细胞存活率下降。MDA检测结果表明，NUF2-KD组细胞的MDA含量显著高于KD-NC组，加入铁死亡抑制剂后MDA含量较NUF2-KD组明显降低（图6B），这表明铁死亡抑制剂能够逆转*NUF2*敲低所导致的细胞MDA含量升高。上述实验结果表明，铁死亡抑制剂Ferrostatin-1能够显著逆转*NUF2*敲低所导致的细胞存活率下降和MDA含量升高，提示*NUF2*敲低可能通过诱导铁死亡来影响细胞的存活和脂质过氧化状态。

**图6:**
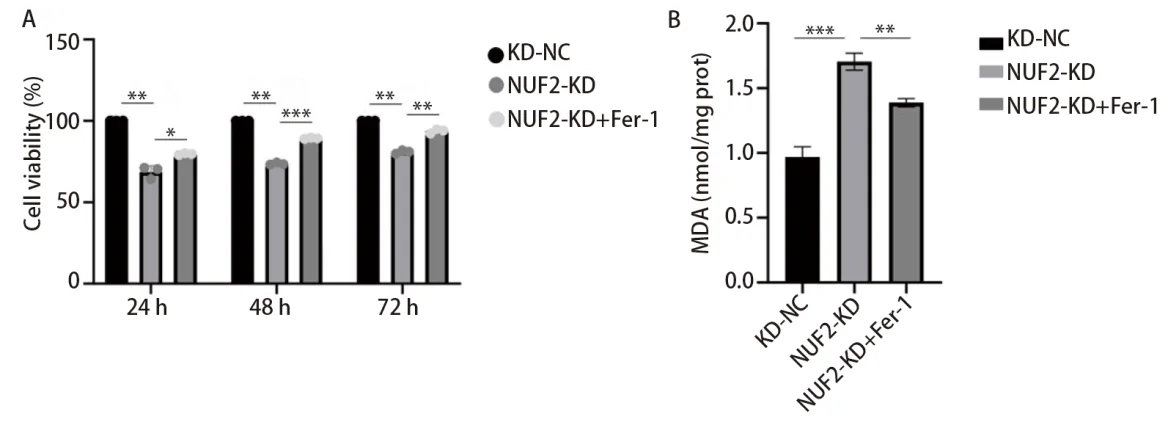
铁死亡抑制剂对NUF2敲低细胞存活率及MDA含量的影响。A：铁死亡抑制剂对NUF2敲低细胞存活率的影响；B：铁死亡抑制剂对NUF2敲低细胞MDA含量的影响。

### 2.5 NUF2在动物体内促进肿瘤生长以及对铁死亡的影响

实验结果（图7A）发现，NUF2-KD组的肿瘤体积小于KD-NC组；与OE-NC组相比，NUF2-OE组裸鼠的肿瘤明显增大。RT-qPCR检测（图7B）和WB（图7C）发现NUF2-KD组的蛋白和mRNA表达水平均降低，过表达后表达水平升高；免疫组化结果（图7D）显示NUF2-KD组的阳性表达率低于KD-NC组，过表达后则相反，证明动物模型构建成功。

**图7:**
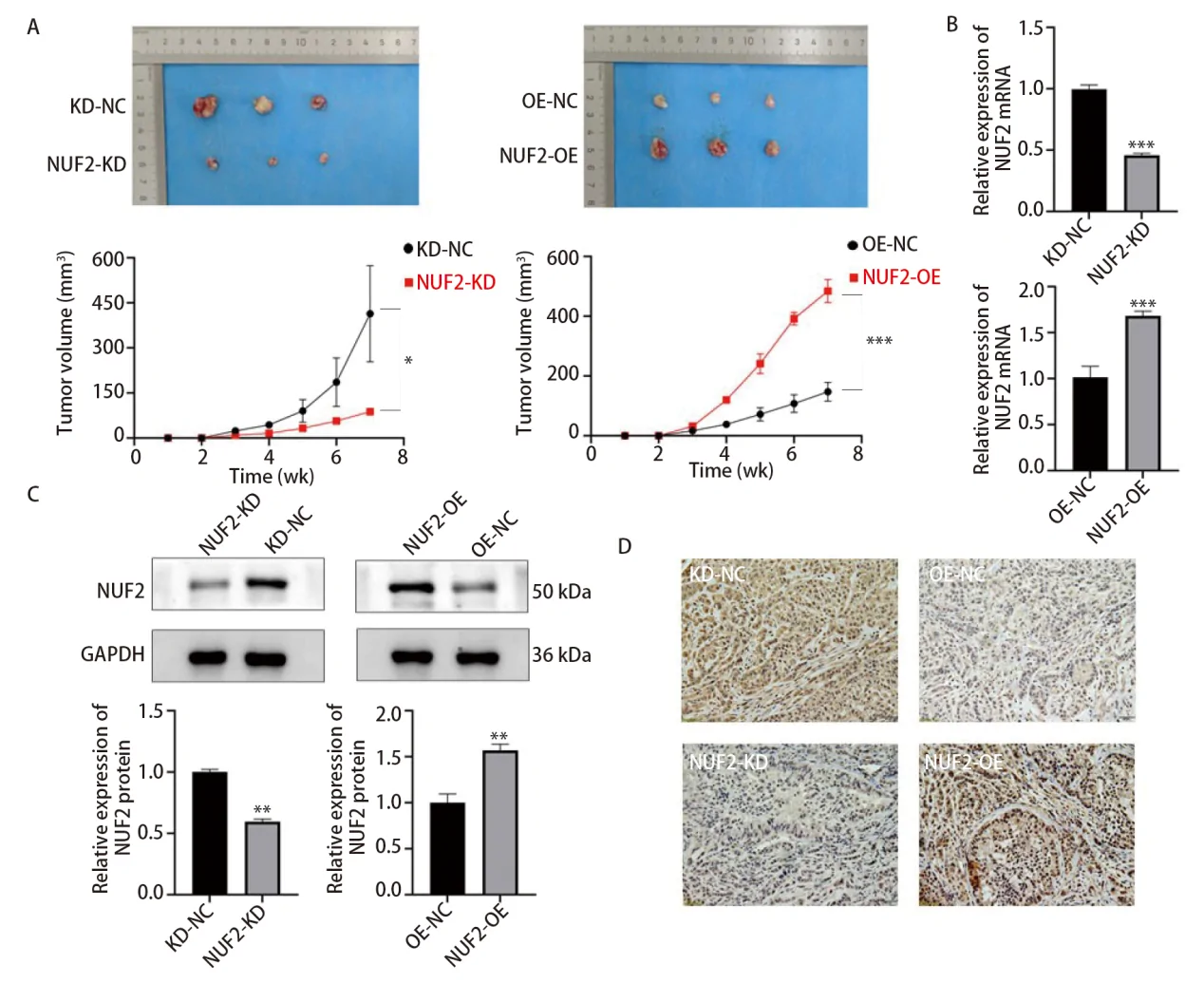
NUF2对裸鼠移植瘤生长的影响。A：NUF2敲低和过表达后裸鼠移植瘤图和肿瘤体积增长曲线；B、C：RT-qPCR和WB检测裸鼠肿瘤组织中NUF2的表达水平；D：IHC检测肿瘤组织中NUF2的阳性表达情况（×200）。

*NUF2*敲低后SLC7A11的蛋白水平降低，而ACSL4的蛋白水平升高；过表达后SLC7A11的蛋白水平升高，而ACSL4的蛋白水平则降低（图8A、8B）。两者的mRNA表达水平变化趋势同蛋白一样（图8C、8D）。免疫组化染色结果显示，ACSL4和SLC7A11表达于细胞膜。NUF2-KD组中SLC7A11的染色强度和阳性细胞比例均低于KD-NC组，ACSL4的染色强度和阳性细胞比例则略高；*NUF2*过表达后实验结果相反（图8E、8F）。上述结果表明，在体内NUF2表达水平影响肿瘤生长，NUF2高表达能够促进NSCLC肿瘤生长，促进SLC7A11的蛋白表达水平而抑制ACSL4的蛋白表达水平，从而抑制NSCLC发生铁死亡。

**图8:**
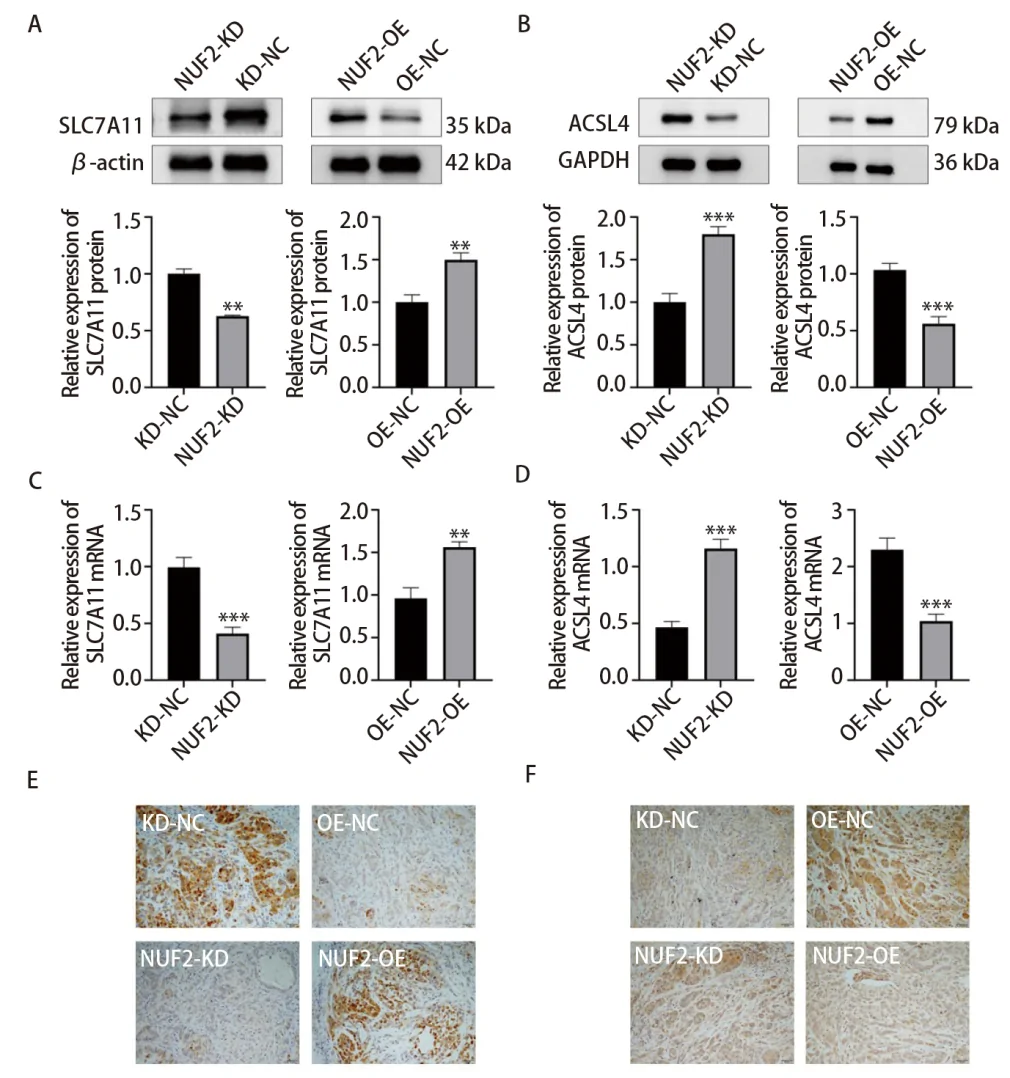
裸鼠移植瘤组织中SLC7A11和ACSL4的表达。A、B：WB检测敲低和过表达NUF2后裸鼠肿瘤组织中SLC7A11和ACSL4的蛋白表达水平；C、D：RT-qPCR检测敲低和过表达NUF2后组织中SLC7A11和ACSL4的mRNA表达水平；E、F：IHC检测裸鼠肿瘤组织中SLC7A11（E）和ACSL4（F）的阳性表达情况（×200）。

## 3 讨论

NSCLC的高异质性、高侵袭性使患者的治疗愈加困难，其死亡率一直居高不下。探索分子标志物及潜在的发生机制可以为NSCLC患者的诊断、治疗和预后提供理论依据。NUF2作为保证染色体正确分离的核心蛋白，当其在癌细胞中异常高表达时，会迫使存在DNA损伤或检查点缺陷的癌细胞强行进入有丝分裂^[16]^。

本研究首先通过GEPIA数据库中肺癌的表达差异分析图以及免疫组化检测的208例肺癌患者的NUF2表达水平发现，肿瘤组织中表达都明显高于正常组织。NUF2的临床关联性分析显示NUF2高表达与患者肿瘤浸润和淋巴结转移有关，有可能作为NSCLC的独立预测指标。为进一步验证上述发现，分别通过体内外实验进行功能探究。细胞实验发现，敲低*NUF2*显著抑制了体外细胞增殖、迁移和侵袭的能力，在小鼠体内同样发现敲低*NUF2*后肿瘤生长受到抑制。由此可见，NUF2在肺癌中发挥促进肿瘤生长和进展的作用，未来可能成为肺癌治疗的分子靶点。

铁死亡是Dixon等^[17]^于2012年首次提出的一种程序性细胞死亡方式，特点是脂质过氧化及ROS的异常蓄积，这些变化共同导致了细胞膜破损，最终诱发细胞死亡。研究^[18]^发现，抑制SLC7A11的表达可抑制肺癌细胞增殖和转移，并诱导铁死亡。而ACSL4高表达可抑制肺癌细胞的增殖和侵袭，同时过氧化产物和ROS积累使肺癌细胞对铁死亡敏感^[19]^。铁死亡在肺癌中具有明确的抑癌作用，肺癌细胞因快速增殖存在铁代谢、氧化还原稳态紊乱的现象，因此诱导肺癌细胞铁死亡有望成为一种新的治疗方法。有研究^[20]^发现，在卵巢癌中USP7可通过去泛素化作用稳定NUF2蛋白，进而调控SLC7A11表达并促进癌症进展。

基于上述背景，本研究初步探讨了NSCLC中NUF2与铁死亡之间的关系，并对相关调控蛋白及铁死亡标志物进行了检测，结果发现，*NUF2*敲低后对铁死亡有抑制作用的SLC7A11表达显著降低，诱导铁死亡发生的蛋白ACSL4表达增加，细胞内MDA、ROS和Fe^2+^的含量明显升高，GSH含量显著降低，且线粒体有明显的皱缩、嵴减少和膜密度增加，提示NSCLC细胞发生了铁死亡。上述结果表明，NUF2可能通过调控SLC7A11与ACSL4表达并进而影响铁死亡。为进一步验证NUF2与铁死亡的关系，本研究使用铁死亡抑制剂逆转了*NUF2*敲低所致的MDA含量升高，表明其能有效抑制*NUF2*敲低引发的脂质过氧化过程。同时，细胞存活率的升高说明铁死亡抑制剂能够保护细胞免受*NUF2*敲低所致的死亡。

综上所述，本研究证实NUF2表达与患者预后不良有关，体内外实验结果表明，NUF2可能通过调控SLC7A11和ACSL4的表达抑制铁死亡从而影响NSCLC的进展。这一机制或将成为调控肺癌的新靶点，为肺癌患者的治疗提供更多的理论依据。

本研究存在局限性。首先，受实际临床收治病例特征、疾病就诊分期及纳入排除标准限制，最终纳入病例中远处转移阳性病例数量偏少，后续将扩大远处转移阳性病例的纳入样本量，均衡各组病例分布，进一步验证本研究相关结论；其次，单一细胞系无法全面反映NSCLC复杂的遗传异质性与表型多样性。因此，本研究结论的普适性仍需在其他NSCLC细胞系中进一步验证。此次研究发现NUF2可能调控SLC7A11与ACSL4的表达，但具体分子机制仍不明确，尤其是定位于细胞核中的NUF2如何调控胞质中SLC7A11与ACSL4的表达。其中涉及的机制可能为，NUF2通过转录因子中介间接调控靶基因转录以及泛素-蛋白酶体系统影响靶蛋白稳定性，这需通过实验进一步验证。最后，本研究在体内实验证实敲低*NUF2*可抑制肿瘤生长，但尚未在动物模型中引入铁死亡抑制剂。针对上述问题，课题组将在后续研究中逐一完善，以期更深入地阐明NUF2调控NSCLC铁死亡的作用机制以及探究其临床转化潜力。
